# The utilization, characteristics, and influencing factors of non-therapeutic traditional Chinese medicine services among middle-aged and older population in Guangzhou, China: a cross-sectional study

**DOI:** 10.3389/fpubh.2026.1772557

**Published:** 2026-03-25

**Authors:** Jie Ren, Jun Hao, Ziqi Jia, Chenxu Zheng, Yaozong Zheng, Zixin Wu, Xiaonan Zhou, Huan Wu, Xintong Ye, Xiaochang Ma, Bin Lin, Lining Yang

**Affiliations:** 1 School of Public Health and Management, Guangzhou University of Chinese Medicine, Guangzhou, China; 2 China Joint Graduate School of Traditional Chinese Medicine, Suzhou, China; 3 School of Marxism, Guangzhou University of Chinese Medicine, Guangzhou, China; 4 First Clinical Medical College, Guangzhou University of Chinese Medicine, Guangzhou, China; 5 Xiyuan Hospital, China Academy of Chinese Medical Sciences, Beijing, China; 6 Second Affiliated Hospital of Shandong University of Traditional Chinese Medicine, Jinan, China

**Keywords:** aging populations, China, influencing factors, service utilization, TCM

## Abstract

**Background:**

As an integral part of the “Healthy China” strategy, healthy aging emphasizes meeting the non-therapeutic health needs of middle-aged and older populations, which is highly consistent with Traditional Chinese Medicine (TCM) concepts. However, empirical studies focusing on the utilization patterns and determinants of such services among the middle-aged and older population remain limited. This study aims to assess the utilization and influencing factors of non-therapeutic TCM services among middle-aged and older people in Guangzhou.

**Methods:**

A cross-sectional survey was conducted from June to October 2025 among 557 individuals aged ≥45 years old in Guangzhou using a structured questionnaire. Chi-square tests and binary logistic regression analyses were used to identify influencing factors.

**Results:**

The overall utilization rate of non-therapeutic TCM services in the past month was 69.7%. Specifically, the utilization rates of health education, preventive health care, chronic disease management, and rehabilitation services were 25.10, 32.70, 28.00, and 30.90%, respectively. Key influencing factors varied by service type: understanding of TCM culture and chronic diseases affected health education utilization; TCM belief, income (≥5,000 yuan/month) and chronic diseases promoted preventive healthcare use; TCM promotion exposure and service satisfaction were associated with chronic disease management; age (≥75 years), regular physical examinations and high-frequency TCM use were associated with rehabilitation service utilization.

**Conclusion:**

Non-therapeutic TCM service utilization is relatively high among middle-aged and older populations in Guangzhou, with type-specific influencing factors. Targeted measures (expanding TCM culture promotion, strengthening chronic disease management, optimizing subsidy policies, and enhancing primary TCM service supply) are recommended to further improve service accessibility.

## 1 Introduction

Traditional Chinese Medicine (TCM) services refer to professional medical services that diagnose and treat patients through the four diagnostic methods (observation, auscultation, inquiry, and pulse-taking) under the guidance of TCM theory (1). Intervention methods mainly include pharmacological treatments (TCM decoctions and proprietary Chinese medicines) and non-pharmacological therapies (acupuncture, tuina, cupping, and guasha) (2). TCM applies to a wide range of populations, particularly demonstrating distinct advantages in managing non-communicable chronic diseases and meeting the health needs of the older population (3–7). Currently, TCM is used in 183 countries and regions around the world (8), and in most cases, it is categorized under traditional medicine, complementary medicine, or integrative medicine (sCIM) (2).

The issuance of the “Healthy China 2030” Planning Outline has promoted the strategic transformation of China’s health care system from disease-centered to health-centered (9). Concurrently, China is experiencing an accelerated aging process, and the health needs of the middle-aged and older population have become increasingly diversified—no longer limited to disease treatment but extending to non-therapeutic services such as preventive health care, health preservation, rehabilitation, and health management (10). “Preventive treatment of diseases”, a core concept of TCM, aligns closely with modern preventive healthcare concepts (11). It emphasizes disease prevention over treatment, which is highly consistent with the health demands of the middle-aged and older population. This concept encompasses a four-stage continuum of care: predisease prevention (health maintenance for asymptomatic individuals); preclinical intervention (early management of subclinical conditions); acute care and complication prevention (treatment and disease progression control); and post-recovery rehabilitation (functional recovery and recurrence prevention). This framework provides comprehensive coverage across the entire health spectrum—from wellness to prognosis. Beyond conventional disease treatment, such services also integrate non-therapeutic TCM interventions, including health education, preventive healthcare, risk screening, chronic disease management, functional rehabilitation, and lifestyle modification (11).

In recent years, a large number of studies have explored the utilization rate of TCM services and influencing factors. TCM service utilization varies significantly across populations and regions (12), ranging from 9 to 94.4% (13, 14). Key influencing factors include age, income, residential location, health behaviors, educational level, disease type, and disease severity (15–17). Additionally, patients’ cognition and trust in TCM have attracted growing research attention (18). However, most existing studies have centered on TCM services applied in therapeutic settings and among patient populations, with insufficient attention devoted to the distinctive characteristics and health-related impacts of non-therapeutic TCM services. This information gap results in insufficient accurate data support for optimizing relevant services, making it challenging to fully meet the personalized health needs of the middle-aged and older population.

By the end of 2024, the older population aged 60 and above in Guangzhou accounted for 19.81% of the total population, with the aging degree continuing to deepen (19). As the birthplace of Lingnan TCM culture, Guangzhou boasts abundant TCM resources and a well-established TCM service system, forming a multi-level TCM service supply network covering Grade A tertiary hospitals to community health service centers (20). The middle-aged and older populations in Guangzhou generally have a high level of cognition and acceptance of TCM, laying a solid foundation for the popularization and promotion of non-therapeutic TCM services (21).

This study adopts a cross-sectional study method, targeting middle-aged and older residents in Guangzhou to investigate their utilization of non-therapeutic TCM services. It analyzes relevant characteristics, including demographic, sociological, and health-related factors, and identifies key determinants of service utilization. The findings aim to provide a scientific basis for Guangzhou’s health authorities to optimize TCM resource allocation and adjust health strategies, thereby better aligning with the “Healthy China” strategy.

## 2 Materials and methods

### 2.1 Study objects

From July 1, 2025, to October 1, 2025, a survey was conducted among middle-aged and older populations in Guangzhou through a combination of online and offline methods using the Wenjuanxing platform. All questionnaire distribution, collection, and data collation were completed within this period. Inclusion criteria: aged ≥45 years, living in the community for ≥6 months, conscious, with basic communication skills, capable of understanding the questionnaire content and completing the survey independently or with the assistance of investigators, providing informed consent and voluntarily participating in the study. Exclusion criteria: suffering from severe cognitive impairment or mental illness and unable to cooperate with the survey, having critical diseases or being hospitalized for a long period, refusing to participate in the survey.

A multi-stage stratified random sampling method was employed for participant recruitment to ensure the representativeness of the study sample. The specific sampling process was as follows: In the first stage, the 11 administrative districts of Guangzhou were stratified into four groups based on the size of the population aged 60 years and older in each district (<100,000; 100,000–200,000; 200,000–300,000; >300,000). One district was randomly selected from each group. In the second stage, two subdistricts were randomly selected from each selected district, and both online and offline surveys were conducted in these subdistricts to ensure that the sample covered administrative districts with different scales of older population.

### 2.2 Sample size calculation

The sample size was calculated using the formula for cross-sectional studies, $n=\frac{\mu_{\frac{\alpha }{2}}^{2}\pi (1-\pi )}{\delta^{2}}$ where n is the minimum required sample size, μ_*α*/2_ = 1.96 when α = 0.05, *π* is the estimated utilization rate (41%, referenced from existing TCM service utilization studies), and *δ* is the allowable error (5%). The minimum sample size was 379 cases. Considering a 20% non-response and invalid response rate, the final sample size was determined to be at least 465 cases.

### 2.3 Questionnaire design

Based on the Anderson Model of Health Service Utilization and relevant previous studies, a questionnaire was developed. Two rounds of expert consultations were conducted to ensure the comprehensiveness, validity, and reliability of the questionnaire. A total of 16 experts with professional titles at or above the intermediate level were invited, with expertise covering TCM clinical practice, medical education, nursing, geriatric health, health policy, health economics, and TCM culture popularization. The questionnaire consisted of five parts: (1) Predisposing characteristics: gender, age, educational level, occupation type, marital status, residential location, living conditions, regular physical examination status, exercise frequency, belief in TCM, understanding of TCM culture, family TCM usage habits, and friend recommendations; (2) Enabling resources: type of medical insurance, monthly income, distance to the nearest TCM-providing medical institution, and community TCM promotion activities; (3) Health needs: self-rated health status, presence of chronic diseases, and comorbidity of chronic diseases; (4) Service utilization: type of service utilized, utilization frequency, type of medical institution visited, trust in doctors, compliance with medical advice, and adjustments to living habits; (5) Health outcomes: satisfaction with TCM services, self-perceived health improvement, willingness to recommend to family and friends, affordability of services, time adequacy, transportation convenience, adequacy of nearby medical resources, and whether health needs were met. All subjective evaluation variables in this study (including attitudes, cognitions, satisfaction, beliefs, and other constructs) were assessed using a standardized single-item 5-point Likert scale. Uniform response categories were applied across all items: scores of 1–5 corresponded to strongly disagree/believe/understand/satisfied to strongly agree/believe/understand/satisfied, with a score of 3 indicating neutrality. Based on the research objectives and conceptual framework, original scores of 1–5 were dichotomized using consistent criteria: scores of 1–3 were coded as 0 (“no,” indicating no positive evaluation), and scores of 4–5 were coded as 1 = (“yes,” indicating positive evaluation).

### 2.4 Theoretical and operational definitions

In this study, TCM services were categorized into therapeutic and non-therapeutic types based on their functional positioning, usage scenarios, and service objectives (22). Therapeutic TCM services refer to intervention measures with the primary purpose of disease diagnosis and clinical treatment. In contrast, non-therapeutic TCM services focus on health maintenance, disease prevention, and quality-of-life enhancement, targeting healthy and sub-healthy populations as well as individuals in the stable or recovery phases of illness (23). The core distinctions between these two categories lie in their service orientation (disease treatment versus health maintenance), target population (diagnosed patients versus healthy, sub-healthy, or rehabilitating individuals), and service objectives (disease elimination versus disease prevention and functional improvement) (23).

This study specifically examines four types of non-therapeutic TCM services: health education, preventive healthcare, chronic disease management, and rehabilitation services. TCM health education services encompass health promotion activities delivered by healthcare professionals, including TCM health education programs, offline lectures on TCM knowledge, and online dissemination of TCM-related videos and graphic materials. TCM preventive healthcare services comprise wellness-oriented interventions such as constitution identification, dietary therapy, and medicinal cuisine, conditioning with appropriate TCM techniques, emotional regulation, and guidance in traditional exercises such as Tai Chi and Baduanjin (24). TCM chronic disease management services provide ongoing preventive and maintenance care for chronic conditions, including condition monitoring, lifestyle intervention, and efficacy evaluation, which are distinct from acute clinical treatment in their focus on disease stabilization and complication prevention (25, 26). TCM rehabilitation services involve functional recovery interventions targeting physical dysfunction resulting from disease, trauma, or aging, with the aim of improving quality of life and restoring daily living capabilities (27).

### 2.5 Statistical analysis

IBM SPSS Statistics 26.0 was used for data analysis. Descriptive statistics were presented as frequencies (n) and constituent ratios (%) for categorical data. Univariate analysis was performed using chi-square tests. Multivariate analysis was conducted using binary logistic regression, with odds ratios (OR) and 95% confidence intervals (CI) used to quantify the strength of associations. *p-*value <0.05 was considered statistically significant.

## 3 Results

### 3.1 Participant characteristics

**Figure 1:**
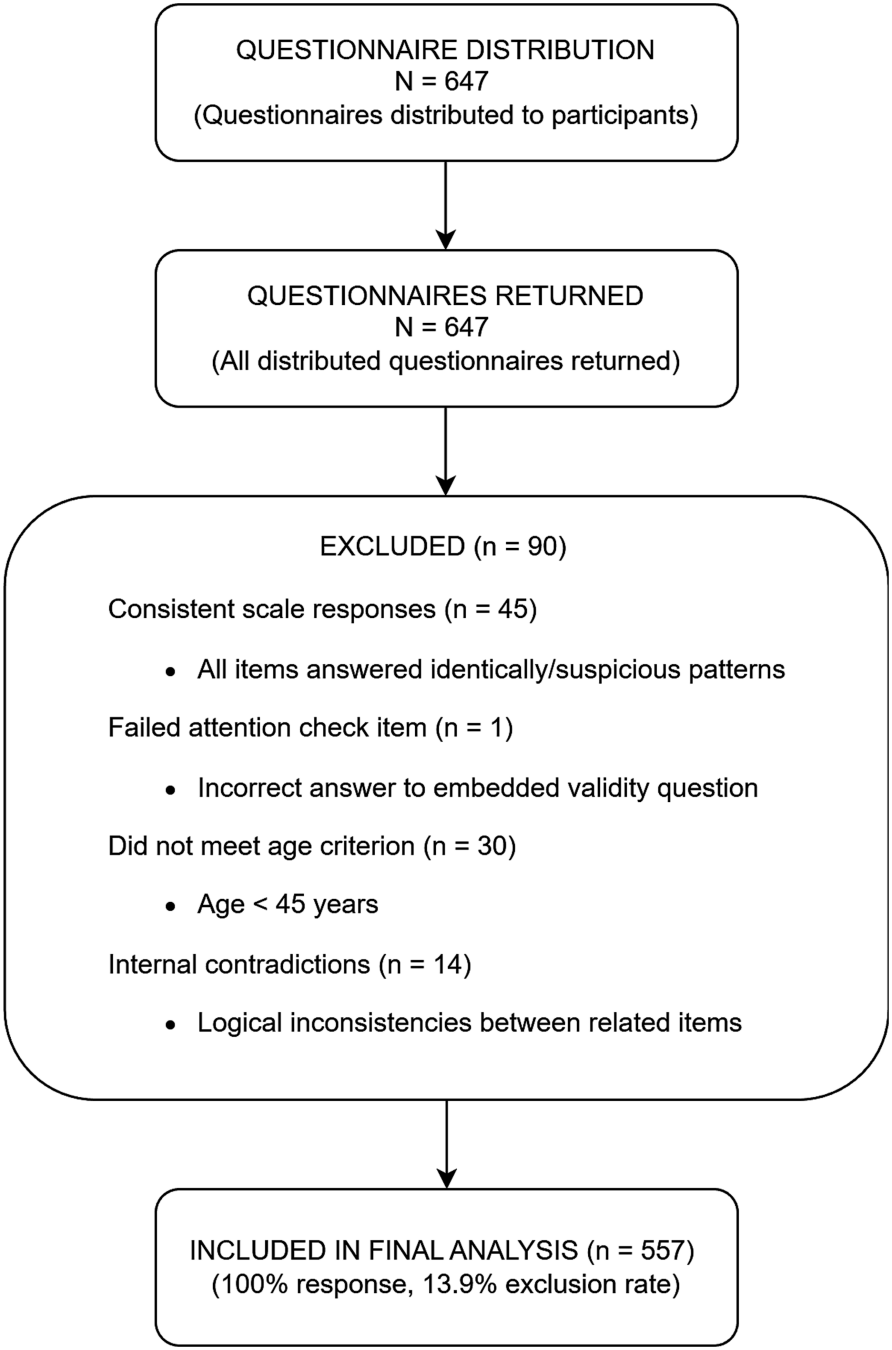
Participant flow diagram.

A total of 647 questionnaires were distributed, with 647 retrieved (response rate: 100%) and 557 valid (validity rate: 86.09%) (Figure 1). Among these 557 valid participants, 10 individuals had never used TCM services, leading to missing data in service utilization and health outcomes variables. Participants with missing values were excluded from statistical analyses. The characteristics of the 557 participants across the dimensions of predisposing characteristics, enabling resources, health needs, service utilization, and health outcomes are presented in Table 1.

**Table 1:** Characteristics of five dimensions.

Variables	Total (*n* = 557)	Health education			Preventive health care			Chronic disease management			Rehabilitation		
		No	Yes	*P*	No	Yes	*P*	No	Yes	*P*	No	Yes	*P*
Gender													
Male	256 (46.00)	194	62	0.646	163	93	0.090	184	72	0.954	172	84	0.363
Female	301 (54.00)	223	78		212	89		217	84		213	88	
Age (Year)													
45–59	267 (47.90)	199	68	0.794	173	94	0.253	199	68	0.301	192	75	0.036*
60–74	214 (38.40)	163	51		153	61		152	62		150	64	
≥75	76 (13.60)	55	21		49	27		50	26		43	33	
Education level													
≤Primary school	129 (23.20)	95	34	0.686	89	40	0.374	92	37	0.994	82	47	0.165
Junior high school	123 (22.10)	88	35		86	37		88	35		94	29	
Senior high school	177 (31.80)	137	40		122	55		128	49		120	57	
≥ college degree	128 (23.00)	97	31		78	50		93	35		89	39	
Occupation type													
Personnel of enterprises and institutions	199 (35.70)	148	51	0.949	125	74	0.223	142	57	0.206	134	65	0.708
Workers and agricultural laborers	157 (28.20)	119	38		108	49		121	36		108	49	
Self employment, freelance work, and others	201 (36.10)	150	51		142	59		138	63		143	58	
Marital status													
Married	47 (8.40)	37	10	0.524	35	12	0.275	35	12	0.693	38	9	0.069
Unmarried	510 (91.60)	380	130		340	170		366	144		347	163	
Residence													
Rural	180 (32.30)	134	46	0.874	124	56	0.587	135	45	0.275	121	59	0.503
Urban	377 (67.70)	283	94		251	126		266	111		264	113	
Living condition													
Living alone	25 (4.50)	21	4	0.370	17	8	0.794	20	5	0.521	20	5	0.111
Core family	395 (70.90)	298	97		269	126		286	109		279	116	
Extended Family	137 (24.60)	98	39		89	48		95	42		86	51	
Regular physical examination													
No	176 (31.60)	134	42	0.638	121	55	0.626	122	54	0.339	135	41	0.008*
Yes	381 (68.40)	283	98		254	127		279	102		250	131	
Exercise frequency													
Low (≤ once every two weeks)	153 (27.50)	114	39	0.850	110	43	0.142	109	44	0.829	100	53	0.081
Medium (once a week)	125 (22.40)	96	29		88	37		88	37		80	45	
High (≥ twice a week)	279 (50.10)	207	72		177	102		204	75		205	74	
Belief in TCM													
No	233 (41.80)	171	62	0.496	170	63	0.016*	161	72	0.197	143	90	0.001*
Yes	324 (58.20)	246	78		205	119		240	84		242	82	
Understand TCM culture													
No	334 (60.00)	263	71	0.010*	241	93	0.003*	246	88	0.286	231	103	0.979
Yes	223 (40.00)	154	69		134	89		155	68		154	69	
Family TCM usage habits													
No	191 (34.30)	147	44	0.410	133	58	0.401	146	45	0.091	131	60	0.844
Yes	366 (65.70)	270	96		242	124		255	111		254	112	
Friend recommendations													
No	276 (49.60)	198	78	0.092	193	83	0.194	188	88	0.043*	187	89	0.489
Yes	281 (50.40)	219	62		182	99		213	68		198	83	
Medical insurance													
Self funded or commercial health insurance	53 (9.50)	39	14	0.821	32	21	0.257	41	12	0.360	34	19	0.410
Medical insurance for urban and rural residents or urban employees	504 (90.50)	378	126		343	161		360	144		351	153	
Monthly income (yuan)													
<2000	121 (21.70)	86	35	0.741	86	35	0.002*	80	41	0.376	81	40	0.797
2000–4,999	249 (44.70)	188	61		182	67		182	67		172	77	
5,000–7,999	150 (26.90)	115	35		89	61		110	40		104	46	
≥8,000	37 (6.60)	28	9		18	19		29	8		28	9	
Distance to the nearest TCM-providing medical institution													
<1 km	62 (11.10)	50	12	0.690	43	19	0.080	51	11	0.088	51	11	0.023*
1–3 km	160 (28.70)	119	41		114	46		117	43		113	47	
3–5 km	197 (35.40)	144	53		119	78		131	66		123	74	
>5 km and unclear	138 (24.80)	104	34		99	39		102	36		98	40	
Community TCM promotion activities													
No	224(40.20)	176	48	0.098	157	67	0.254	176	48	0.005*	158	66	0.553
Yes	333 (59.80)	241	92		218	115		225	108		227	106	
Self-rated health status													
Poor	86 (15.40)	61	25	0.658	60	26	0.846	62	24	0.882	53	33	0.227
Fair	262 (47.00)	198	64		174	88		191	71		182	80	
Good	209 (37.50)	158	51		141	68		148	61		150	59	
Chronic diseases													
No	52 (9.30)	50	2	<0.001*	44	8	0.005*	48	4	0.001*	48	4	<0.001*
Yes	505 (90.70)	367	138		331	174		353	152		337	168	
Comorbidity of chronic diseases													
No	150 (26.90)	131	19	<0.001*	102	48	0.837	123	27	0.001*	123	27	<0.001*
Yes	407 (73.10)	286	121		273	134		278	129		262	145	
Utilization frequency													
Low (≤ 3 times a month)	99 (18,10)	78	21	0.514	64	35	0.355	81	18	0.030*	83	16	0.001*
Medium (4–7 times a month)	205 (37.50)	149	56		131	74		138	67		131	74	
High (≥ 8 times per month)	243 (44.40)	180	63		170	73		172	71		161	82	
Type of medical institution visited													
Primary level	76 (13.90)	66	10	0.008*	50	26	0.338	61	15	0.441	62	14	0.016*
County level	113 (20.70)	88	25		72	41		77	36		66	47	
Municipal level	124 (22.70)	81	43		76	48		89	35		84	40	
Provincial level	192 (35.10)	138	54		138	54		135	57		136	56	
other	42 (7.70)	34	8		29	13		29	13		27	15	
Trust in doctors													
No	232 (42.40)	166	66	0.189	154	78	0.882	168	64	0.678	152	80	0.189
Yes	315 (57.60)	241	74		211	104		223	92		223	92	
Compliance with medical advice													
No	163 (29.80)	117	46	0.359	105	58	0.455	110	53	0.177	108	55	0.451
Yes	384 (70.20)	290	94		260	124		281	103		267	117	
Adjustments to living habits													
No	186 (34.00)	136	50	0.620	123	63	0.831	124	62	0.073	119	67	0.098
Yes	361 (66.00)	271	90		242	119		267	94		256	105	
Satisfied with TCM services													
No	154 (28.20)	111	43	0.435	103	51	0.961	120	34	0.037*	102	52	0.464
Yes	393 (71.80)	296	97		262	131		271	122		273	120	
Self-perceived health improvement													
General and below	203 (37.10)	156	47	0.315	131	72	0.402	140	63	0.317	132	71	0.172
Significant and above	344 (62.90)	251	93		234	110		251	93		243	101	
willingness to recommend to family and friends													
No	161 (29.40)	113	48	0.144	109	52	0.755	112	49	0.522	110	51	0.940
Yes	386 (70.60)	294	92		256	130		279	107		265	121	
Able to afford TCM services													
No	258 (47.20)	195	63	0.552	183	75	0.049*	179	79	0.304	170	88	0.205
Yes	289 (52.80)	212	77		182	107		212	77		205	84	
Having sufficient time to receive TCM services													
No	190 (34.70)	140	50	0.778	135	55	0.117	137	53	0.813	132	58	0.736
Yes	357 (65.30)	267	90		230	127		254	103		243	114	
Convenient transportation													
No	186 (34.00)	144	42	0.246	128	58	0.457	133	53	0.993	123	63	0.380
Yes	361 (66.00)	263	98		237	124		258	103		252	109	
Adequate medical resources nearby													
No	229 (41.90)	166	63	0.383	152	77	0.882	165	64	0.802	158	71	0.851
Yes	318 (58.10)	241	77		213	105		226	92		217	101	
Meet health needs													
No	224 (41.00)	168	56	0.791	144	80	0.313	162	62	0.717	148	76	0.297
Yes	323 (59.00)	239	84		221	102		229	94		227	96	

* = significantly associated factors.

### 3.2 Utilization status

Among the 557 participants, 547 (98.20%) had prior experience with TCM services. Within the past month, 426 (76.50%) had used TCM services, and 388 (69.70%) had utilized non-therapeutic TCM services. Among the non-therapeutic service types, preventive health care services were the most commonly used (182 cases, 32.70%), followed by rehabilitation services (172 cases, 30.90%) and chronic disease management services (156 cases, 28.00%), while health education services had the lowest utilization rate (140 cases, 25.10%). In terms of intervention methods, participants showed a greater preference for appropriate TCM technologies (233 cases, 41.80%) compared to oral Chinese medicines (181 cases, 32.50%). Detailed data are presented in Table 2.

**Table 2:** The utilization of non-therapeutic TCM services.

Main category	Classification	Frequency (n)	Composition ratio (%)
Utilization	Previously used TCM services	547	98.20
	Used TCM services in the past month	426	76.50
	Used non-therapeutic TCM services in the past month	388	69.70
Service type	TCM health education services	140	25.10
	TCM preventive health care services	182	32.70
	TCM chronic disease management services	156	28.00
	TCM rehabilitation services	172	30.90
Intervention method	Oral administration of TCM	181	32.50
	Appropriate techniques for TCM	233	41.80

### 3.3 Reasons for prioritizing TCM

Among the 557 participants, 533 (95.69%) indicated they would prioritize TCM when having health needs. Belief in TCM was the primary reason for choosing TCM (247 cases, 44.3%), followed by good therapeutic efficacy (225 cases, 40.4%), high quality of Chinese medicines (209 cases, 37.5%), and excellent medical skills of TCM practitioners (208 cases, 37.3%). Regarding disease severity, 28.5% of participants would choose TCM for severe diseases, while 16.5% would prioritize TCM for mild conditions. Twenty-four participants (4.31%) stated they would not prioritize TCM, with the main reasons being the long treatment cycle of Chinese medicines (12 cases, 2.2%) and the inconvenience of administration (10 cases, 1.8%). Detailed data are presented in Table 3.

**Table 3:** Reasons for prioritizing TCM.

Classification	Reason	Frequency (*n*)	Composition ratio (%)
Prioritize TCM	Believe in TCM	247	44.30
	Good effect	225	40.40
	Good quality of Chinese herbal medicine	209	37.50
	Good technical skills of doctors	208	37.30
	Good service attitude of doctors	190	34.10
	Convenient transportation	178	32.00
	Cheap	178	32.00
	Severe illness	159	28.50
	Minor side effects of Chinese herbal medicine	159	28.50
	Mild illness	92	16.50
Do not prioritize TCM	Long cycle of TCM treatment	12	2.20
	Difficulty in taking TCM	10	1.80
	Relatively high price	7	1.30
	Poor effect	6	1.10
	Significant side effects of Chinese herbal medicine	6	1.10
	Poor quality of Chinese herbal medicine	5	0.90
	Do not believe TCM	5	0.90
	Mild illness	2	0.40
	Severe illness	2	0.40
	Poor service attitude of doctors	2	0.40
	Poor technical skills of doctors	1	0.20
	Poor transportation	1	0.20

### 3.4 Influencing factors

Univariate analysis results (Table 1) showed that utilization of TCM health education services differed significantly by understanding of TCM culture (*p* = 0.010), presence of chronic diseases (*p* < 0.001), comorbidity of chronic diseases (*p* < 0.001), and type of medical institution visited (*p* = 0.008). Utilization of TCM preventive health care services varied by belief in TCM (*p* = 0.016), understanding of TCM culture (*p* = 0.003), income level (*p* = 0.002), presence of chronic diseases (*p* = 0.005), and service affordability (*p* = 0.049). Factors influencing utilization of TCM chronic disease management services included friend recommendations (*p* = 0.043), community TCM promotion (*p* = 0.005), presence of chronic diseases (*p* = 0.001), comorbidity of chronic diseases (*p* = 0.001), utilization frequency (*p* = 0.030), and satisfaction with TCM services (*p* = 0.037). Factors affecting utilization of TCM rehabilitation services included age (*p* = 0.036), regular physical examinations (*p* = 0.008), belief in TCM (*p* = 0.001), distance to medical institutions (*p* = 0.023), presence of chronic diseases (*p* < 0.001), comorbidity of chronic diseases (*p* < 0.001), utilization frequency (*p* = 0.001), and type of medical institution visited (*p* = 0.016). Variables with *p* < 0.05 in univariate analysis were included in multivariate analysis.

Binary logistic multivariate regression analysis was performed using the backward LR method (Table 4). The results indicated that middle-aged and older individuals who understood TCM culture (OR = 1.174, 95%CI: 1.150–2.555, *p* = 0.008), had chronic diseases (OR = 4.824, 95%CI: 1.057–22.018, *p* = 0.042), had comorbid chronic diseases (OR = 1.83, 95%CI: 1.029–3.254, *p* = 0.040), and sought medical care at provincial/municipal hospitals (OR = 1.762, 95%CI: 1.124–2.763, *p* = 0.014) were more likely to use TCM health education services. Compared to those who did not believe in TCM or understand TCM culture, individuals who believed in TCM (OR = 1.654, 95%CI: 1.126–2.430, *p* = 0.010) and understood TCM culture (OR = 1.532, 95%CI: 1.055–2.225, *p* = 0.025) were more inclined to use TCM preventive health care services. Those with chronic diseases (OR = 3.632, 95%CI: 1.619–8.146, *p* = 0.002) and monthly income ≥5,000 yuan (OR = 1.949, 95%CI: 1.329–2.860, *p* = 0.001) also had a higher likelihood of utilizing TCM preventive health care services. Presence of chronic diseases (OR = 3.885, 95%CI: 1.338–11.276, *p* = 0.013), exposure to community TCM promotion activities (OR = 1.637, 95%CI: 1.091–2.457, *p* = 0.017), and satisfaction with TCM services (OR = 1.815, 95%CI: 1.163–2.834, *p* = 0.009) were associated with increased utilization of TCM chronic disease management services. The probability of TCM rehabilitation service utilization among individuals aged ≥75 years was twice that of those aged 45–59 years (OR = 2.119, 95%CI: 1.216–3.692, *p* = 0.008). Individuals who underwent regular physical examinations (OR = 1.727, 95%CI: 1.121–2.659, *p* = 0.013) and had chronic diseases (OR = 3.183, 95%CI: 1.080–9.384, *p* = 0.036) were more likely to use TCM rehabilitation services. Compared to those seeking care at primary hospitals, individuals who visited district/county-level hospitals (OR = 2.352, 95%CI: 1.130–4.896, *p* = 0.022) had a higher likelihood of utilizing TCM rehabilitation services. Additionally, individuals who used TCM services ≥4 times per month were more than twice as likely to use TCM rehabilitation services as those with utilization frequency ≤3 times per month (OR = 2.295, 95%CI: 1.255–4.197, *p* = 0.007).

**Table 4:** Multivariate analysis of influencing factors.

Service type	Variables	*B*	SE	Wald	*P*	Exp(B)	95% CI
TCM health education services	Understand TCM culture						
	No					Ref	
	Yes	0.539	0.20	6.99	0.008*	1.714	1.150–2.555
	Chronic diseases						
	No					Ref	
	Yes	1.574	0.78	4.126	0.042*	4.824	1.057–22.018
	Comorbidity of chronic diseases						
	No					Ref	
	Yes	0.604	0.29	4.236	0.040*	1.83	1.029–3.254
	Type of medical institution visited			6.793	0.0303*		
	Primary or county hospitals					Ref	
	Provincial or municipal hospitals	0.567	0.23	6.101	0.014*	1.762	1.124–2.763
	Other	0.058	0.45	0.017	0.896	1.06	0.442–2.543
TCM preventive health care services	Belief in TCM						
	No					Ref	
	Yes	0.503	0.196	6.568	0.010*	1.654	1.126–2.430
	Understand TCM culture						
	No					Ref	
	Yes	0.427	0.19	5.019	0.025*	1.532	1.055–2.225
	Chronic diseases						
	No					Ref	
	Yes	1.29	0.412	9.793	0.002*	3.632	1.619–8.146
	Monthly income (yuan)						
	<5,000					Ref	
	≥5,000	0.668	0.195	11.66	0.001*	1.949	1.329–2.860
TCM chronic disease management services	Chronic diseases						
	No					Ref	
	Yes	1.357	0.544	6.229	0.013*	3.885	1.338–11.276
	Community TCM promotion activities						
	No					Ref	
	Yes	0.493	0.207	5.67	0.017*	1.637	1.091–2.457
	Satisfied with TCM services						
	No					Ref	
	Yes	0.596	0.227	6.883	0.009*	1.815	1.163–2.834
	Utilization frequency						
	Low					Ref	
	Medium and high	0.524	0.289	3.297	0.069	1.689	0.959–2.973
TCM rehabilitation services	Age (Year)			7.025	0.030*		
	45–59					Ref	
	60–74	0.201	0.213	0.889	0.346	1.223	0.805–1.857
	≥75	0.751	0.283	7.025	0.008*	2.119	1.216–3.692
	Regular physical examination						
	No					Ref	
	Yes	0.546	0.22	6.143	0.013*	1.727	1.121–2.659
	Chronic diseases						
	No					Ref	
	Yes	1.158	0.552	4.405	0.036*	3.183	1.080–9.384
	Type of medical institution visited			7.085	0.029*		
	Primary					Ref	
	County level	0.855	0.374	5.227	0.022*	2.352	1.130–4.896
	Provincial, municipal hospitals or others	0.332	0.338	0.962	0.327	1.394	0.718–2.705
	Utilization frequency						
	Low					Ref	
	Medium and high	0.831	0.308	7.282	0.007*	2.295	1.255–4.197

Sensitivity analysis was performed using the enter method (including all initial variables) to test the sensitivity of the results to model specifications. The results showed that the consistency rate of the significantly influencing factors screened by the two regressions was 81.25%, and the fluctuation range of OR values was ≤10%. Three variables that were significant in the original regression became non-significant in the sensitivity analysis, with *p-*values of 0.061, 0.156, and 0.05, respectively. The reason for this fluctuation difference can be attributed to the different specifications of the two models: the original backward LR regression effectively controlled potential collinearity by gradually excluding redundant variables, while the enter method was used in the sensitivity analysis to include all initial variables without excluding redundant variables, which may lead to minor collinearity. However, the above three variables with differences did not have a substantial impact on the effect intensity and direction of the other significant variables. Overall, the multivariable analysis results of this study exhibit a certain degree of robustness.

## 4 Discussion

Non-therapeutic TCM services not only play a crucial role in meeting the health needs of the contemporary middle-aged and older population but also align with the key directions of the “Healthy China” strategy. This study found that 98.20% of participants had prior experience with TCM services, which is significantly higher than the utilization rates reported among older populations in Hunan (61.90%) and Zhejiang (29.38%) (28, 29). The monthly TCM service utilization rate in this study (76.50%) was also higher than that of home-based older care groups in Hangzhou (48.72%) and Taiwan (7.3%) (30, 31). These discrepancies indicate that the middle-aged and older population in Guangzhou has a high level of acceptance of TCM, with TCM use being widespread. The utilization rate of non-therapeutic TCM services (69.70%) was slightly lower than that of TCM services. In contrast, the utilization rate of community non-therapeutic health services among older individuals aged ≥60 years in Shaanxi was 32.31% (32), while the consumption rate of dietary Chinese medicines in Cantonese-speaking areas of Guangzhou over the past 12 months reached 74.7% (33). This suggests that TCM usage habits have been integrated into the daily lives of Guangzhou residents, extending beyond TCM services provided by medical institutions.

Regarding intervention methods, the utilization rate of appropriate TCM technologies (41.80%) was higher than that of oral Chinese medicines (32.50%) in this study. A previous survey of chronic disease patients in China showed that 73.11% of patients using TCM therapies opted for Chinese medicines, significantly higher than the utilization rate of appropriate TCM technologies such as acupuncture, moxibustion, and cupping (2). However, among first-time stroke inpatients, the utilization rate of acupuncture was 79.7%, while that of oral Chinese medicines was only 5.1% (34). These differences were mainly related to variations in utilization purposes driven by population characteristics.

A total of 95.69% of participants reported prioritizing TCM for health needs. During the COVID-19 pandemic, the acceptance rate of TCM among asymptomatic infected individuals in Shanghai was 91.35% (35), which is generally consistent with the findings of this study, though population differences should be considered. Among Chinese immigrants in Canada, 48.8% chose TCM as a preventive measure, while only 20.8% believed that Western medicine could effectively prevent infections (36). In contrast, a study on medical-seeking tendencies based on the Taiwan Biobank found that 50.8% of participants preferred Western medicine treatment, and only 10.4% preferred TCM (37). These significant differences are mainly associated with variations in regional and socio-cultural backgrounds (15). Regarding reasons for choosing TCM, good therapeutic efficacy has been supported by previous studies (35, 38). Individuals who did not accept TCM cited time constraints and inconvenience as the main barriers, whereas previous studies identified lack of physician recommendations and concerns about side effects as key factors (35, 38). This indicates that the middle-aged and older population in Guangzhou is more concerned with practical barriers during TCM utilization.

The utilization rate of TCM preventive health care services was 32.70%, slightly higher than that of urban residents in Chongqing (27.3%) (39) and similar to that of migrant older populations in China (32.64%) (40). A study in Shanghai reported that 48.3% of high-risk diabetes populations used such services within the past year (41). Belief in TCM, understanding of TCM culture, presence of chronic diseases, and monthly income ≥5,000 yuan were identified as factors associated with TCM preventive health care service utilization, which is consistent with previous findings (2, 29, 35, 42). TCM possesses inherent cultural attributes; individuals who understand TCM culture were associated with a greater likelihood of embracing the concept of “preventive treatment of disease” and were more likely to believe in the effectiveness of TCM interventions for disease prevention. Chronic disease patients typically have a certain understanding of their conditions and more proactive engagement in preventive measures to avoid disease progression or comorbidities. Economic status was related to payment capacity—individuals with lower monthly incomes tended to allocate limited resources to basic living needs and existing health problems, which corresponded to weaker willingness and capacity to pay for non-essential preventive health care services.

The utilization rate of TCM rehabilitation services was 30.90%, lower than the utilization rate of appropriate TCM technologies among older rehabilitation patients (41.19%) (43). Presence of chronic diseases and age ≥75 years were key factors associated with TCM rehabilitation service utilization. A survey in Jiangsu found that age ≥65 years was associated with higher TCM service utilization rates (13). Individuals aged ≥75 years, belonging to the advanced older group, often experience physical function decline and long-term chronic diseases, which were correlated with a higher risk of sequelae or functional impairments, and thus corresponded to greater rehabilitation needs. Individuals who undergo regular physical examinations showed a higher tendency to use TCM rehabilitation services, as those requiring rehabilitation typically have poor health status and may receive physician recommendations for regular physical examinations to assess their condition. Compared to those seeking medical care at primary hospitals, individuals who visited district/county-level hospitals were more inclined to use TCM rehabilitation services—a finding consistent with a survey of older dementia patients (44). Currently, the coverage rate of TCM services in primary medical institutions in Guangzhou exceeds 80%, but the supply capacity of systematic and standardized rehabilitation services remains limited. Consequently, individuals with rehabilitation needs were more likely to seek medical care at higher-level hospitals. This study also found that individuals who used TCM services ≥4 times per month showed a higher probability of using TCM rehabilitation services. Previous studies have similarly noted that chronic disease patients who visited medical institutions multiple times per week had higher TCM utilization rates (2). Additionally, a high frequency of complementary and alternative medicine (CAM) utilization (40.0%) was observed among Parkinson’s patients receiving rehabilitation treatment, compared to 22.9% for CAM utilization unrelated to Parkinson’s disease (45). High-frequency medical service utilization is typically associated with persistent and complex health problems, which aligns with the health characteristics of the older population. Regular rehabilitation treatment was correlated with significant improvements in motor symptoms and quality of life (45), and trust accumulation and behavioral inertia resulting from symptom improvement may reduce the threshold for utilizing rehabilitation services.

The prevalence of chronic diseases among middle-aged and older populations in Guangzhou was 90.70%, yet the utilization rate of chronic disease management services was only 28.00%. Presence of chronic diseases was an important factor related to the utilization of TCM chronic disease management services. Previous studies have reported TCM service utilization rates among chronic disease patients ranging from 14.0 to 43.3% (46–52), with differences mainly linked to variations in disease types and service utilization purposes. Satisfaction with TCM services and exposure to community TCM promotion activities were also associated with increased utilization of TCM chronic disease management services, which is consistent with previous findings (53, 54). Chronic disease patients who are satisfied with TCM services demonstrated a higher tendency to continue using TCM. Common chronic diseases such as hypertension and diabetes often have no obvious early symptoms, which is related to insufficient patient awareness of the importance of disease management. A previous study found that the awareness rate of regular medication among rural hypertension patients in Heilongjiang was only approximately 60%, and the awareness rate of lifelong medication was even lower (52). However, increased access to health information was correlated with a higher likelihood of TCM utilization (2).

TCM health education services had the lowest utilization rate (25.10%). Understanding of TCM culture was associated with higher acceptance of TCM health education. Studies among Chinese immigrant groups have emphasized the importance of cultural adaptation for health education programs (55), and a stronger cultural identity was related to greater trust in health knowledge. TCM’s integration into daily life also contributed to a lower threshold for accepting TCM health education. Presence of chronic diseases and comorbidities was also associated with the utilization of health education services. A study of cardiovascular disease patients found that 50.3% of participants were interested in health education (56). Additionally, patients with comorbid chronic diseases typically have poorer health status and prognosis, requiring comprehensive management, and physicians may provide more health education to this group. Middle-aged and older individuals who sought care at provincial/municipal hospitals were more likely to use TCM health education services. As regional medical centers, provincial/municipal hospitals offer more professional and systematic TCM-related services compared to lower-level hospitals. Preference for authoritative health information was also linked to greater demand for health education at higher-level medical institutions among the middle-aged and older population (57).

This study has some limitations. First, as a cross-sectional study using self-reported questionnaires, it is inherently subject to recall bias and cannot establish causal relationships. To minimize the impact of information bias, we adopted a standardized questionnaire design. However, certain information bias may still exist, which needs to be considered when interpreting the results. Further longitudinal studies are required to verify the findings and establish potential causal relationships. Second, despite the adoption of scientific sampling methods, potential selection bias may still exist due to the combination of online surveys — for example, online respondents may be more familiar with internet operation, which could lead to certain deviations in sample characteristics. Third, all subjective evaluation variables were measured using single-item scales. Although this approach helps control questionnaire length while preserving data quality in large-scale surveys, it precludes the assessment of internal consistency reliability and may not fully capture the complexity of the constructs. Furthermore, dichotomization of subjective scale scores results in the loss of some detailed information, which may not fully reflect the subtle differences in the subjective attitudes of respondents. Fourth, the study was limited to Guangzhou, and differences in regional and cultural backgrounds may limit the generalizability of the findings. Specifically, most associations identified in this study are consistent with previous studies and may be replicable across similar populations. However, the high baseline engagement and utilization rate observed in this study are context-specific, shaped by Guangzhou’s unique regional culture, residents’ TCM usage habits, and local health service supply characteristics, which may not be directly generalizable to other regions with different cultural and health system backgrounds. In the future, large-scale multi-population and multi-regional surveys should be conducted to better understand the overall trends in non-therapeutic TCM service utilization and provide more targeted references for policy formulation.

## 5 Conclusion

This study investigates the utilization status and influencing factors of non-therapeutic TCM services among middle-aged and older populations in Guangzhou. The overall utilization rate of non-therapeutic TCM services was 69.70%, with utilization rates of 25.10, 32.70, 28.00, and 30.90% for health education, preventive health care, chronic disease management, and rehabilitation services, respectively. Key influencing factors include trust in TCM, understanding of TCM culture, presence of chronic diseases, type of medical institution visited, income level, community TCM promotion, satisfaction with TCM services, utilization frequency, age, and regular physical examination status. Based on these context-specific findings, the following tentative recommendations are proposed for future reference, given that this study does not evaluate intervention effects: (1) Consider expanding the promotion of TCM knowledge and culture through integrated online and offline approaches, particularly in regions with similar cultural backgrounds to Guangzhou; (2) Explore leveraging TCM’s holistic concept to strengthen comprehensive management of comorbid chronic diseases, with reference to the characteristics of the middle-aged and older population in this study; (3) Future policy efforts could consider formulating targeted subsidy policies for low-income groups to reduce potential barriers to accessing preventive health care services, which may be adjusted according to regional economic and cultural differences; (4) Consider enhancing the supply capacity of rehabilitation services in primary medical institutions in Guangzhou to improve local service accessibility, with caution in generalizing this approach to other regions.

## Data Availability

The original contributions presented in the study are included in the article/supplementary material, further inquiries can be directed to the corresponding author/s.
